# Intraoperative and Postoperative Outcomes of Pfannenstiel and Midline Skin Incisions in Placenta Accreta Spectrum Disorders: Single-Center Experience

**DOI:** 10.3390/medicina60071102

**Published:** 2024-07-05

**Authors:** Hulya Kandemir, Emine Kirtis, Gul Alkan Bulbul, Selen Dogan, Inanc Mendilcioglu, Cem Yasar Sanhal, Mehmet Sakinci, Nasuh Utku Dogan

**Affiliations:** The Department of Obstetrics and Gynecology, Akdeniz University Faculty of Medicine, Antalya 07070, Türkiye; dr.hulya.kandemir@gmail.com (H.K.); edogru07@hotmail.com (E.K.); gulalkan15@hotmail.com (G.A.B.); drsalben@hotmail.com (S.D.); imendilcioglu@gmail.com (I.M.); cemsanhal@yahoo.com (C.Y.S.); mehmetsakinci@hotmail.com (M.S.)

**Keywords:** antepartum hemorrhage, cesarean hysterectomy, emergency surgery, midline skin incision, Pfannenstiel incision, placenta accreta spectrum

## Abstract

*Background:* We compared Pfannenstiel and midline skin incisions for cesarean hysterectomy in women with confirmed Placenta Accreta Spectrum Disorders. *Aims:* A retrospective cohort study was conducted to evaluate the outcomes of Pfannenstiel and midline skin incisions in women undergoing cesarean section hysterectomy for suspected placenta accreta at Akdeniz University Hospital between January 2010 and February 2022. Histopathological confirmation was obtained for all cases. Demographic, perioperative, and postoperative data, along with neonatal outcomes, were extracted from the hospital’s electronic database. Possible complaints related to the incision site or other issues (e.g., vaginal dryness or sexual life) were identified through telephone interviews. Subjects were stratified into Pfannenstiel and midline incision cohorts, with subsequent data comparison. *Results:* Data from 67 women with a histopathologically confirmed PAS diagnosis were analyzed. Of these, 49 (73.1%) underwent Pfannenstiel incision, and 18 (26.9%) had a midline skin incision. Incisions were based on the surgeon’s experience. Pfannenstiel incision was more common in antepartum hemorrhage, preoperative hemorrhage, and emergency surgery (*p* = 0.02, *p* = 0.014, *p* = 0.002, respectively). Hypogastric artery ligation occurred in 30 cases (61.2%) in the Pfannenstiel group but none in the midline group. Cosmetic dissatisfaction and sexual problems were more prevalent in the midline group (*p* < 0.05, all). Preoperative and postoperative blood parameters, transfused blood products, and neonatal outcomes were similar between the two groups. *Conclusions:* Relaparotomy, bladder injury, blood loss, and need for blood transfusion were more prevalent in the Pfannenstiel group, while greater dissatisfaction with the incision was observed in the midline incision group. Midline incision seems to be more favorable in patients with Placenta Accreta Spectrum (PAS). Patients may be informed regarding the worse cosmetic outcomes and possible sexual problems related to vaginal dryness when midline laparotomy is planned. But before opting for a Pfannenstiel incision, patients should receive comprehensive information regarding the potential risks of relaparotomy and bladder injury.

## 1. Introduction

Placenta Accreta Spectrum (PAS) is a histopathological diagnosis that results in the abnormal adhesion or invasion of trophoblasts into or beyond the myometrium [1]. The prevalence is 0.79–3.11 per 1000 births [2]. The prevalence is increasing due to increasing cesarean rates [3] and the most important risk factor is placenta previa after previous cesarean delivery [4]. The diagnostic sensitivity and specificity of grayscale and color Doppler USG were reported as 90.72% and 96.94% [5]. It has maternal morbidity such as heavy blood loss, emergency cesarean, peripheral tissue injury, massive transfusion requirement [6], and mortality of up to 7% [7].

No standardized surgical method has been reported for PAS surgery, even in centers of excellence. Previous studies have explored various approaches to reduce maternal mortality and morbidity and improve neonatal outcomes, including antenatal diagnoses and management by a multidisciplinary team [8], planned cesarean delivery, preoperative ureteral stent placement, hypogastric artery ligation [9], balloon application to the aorta or hypogastric artery, arterial embolization [10,11], anemia treatment, medications to reduce blood loss, subtotal hysterectomy, and leaving the placenta in situ [12]. The comparison of transverse and vertical skin incisions on perioperative and postoperative outcomes in cesarean hysterectomy was conducted for the first time in 2020 [13].

In the present study, we aimed to evaluate surgical complications, surgical duration, postpartum complications, and neonatal outcomes associated with Pfannentiel and midline skin incisions in PAS surgery at our hospital. Through this study, we intended to demonstrate the impact of different skin incision types on clinical outcomes. 

## 2. Materials and Methods

### 2.1. Study Design and Population

**Figure 1:**
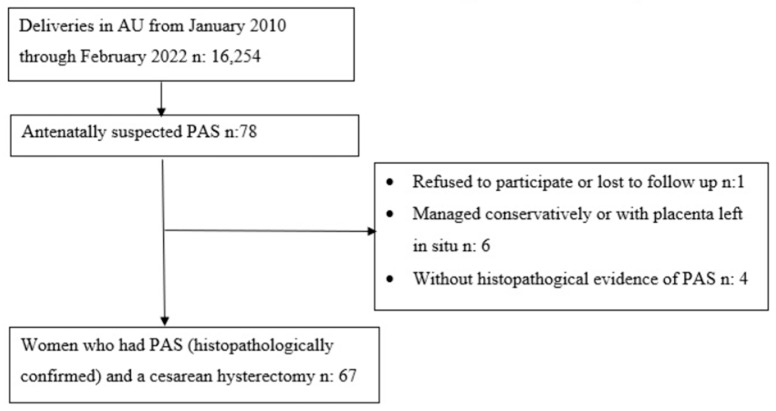
Flowchart of inclusion process of women with history of Placenta Accreta Spectrum. AU, Akdeniz University; PAS, Placenta Accreta Spectrum; n, Number of Patients.

This study is a single-center retrospective cohort study. Patients with suspected PAS during the antenatal period at Akdeniz University Hospital (a tertiary center experienced in PAS surgery) between January 2010 and February 2022, who underwent cesarean hysterectomy and whose diagnosis was confirmed histopathologically, were included. During this period, 16,254 deliveries occurred in our hospital and 78 women were diagnosed with ‘accreta suspicion’ (during antenatal evaluation or evaluations under emergency conditions such as hemorrhage or pain). The diagnosis of ‘accreta suspicion’ was made by experienced maternal–fetal medicine specialists in the perinatology unit of our hospital. All patients were evaluated with USG and, if necessary, with color Doppler USG, 3D USG, and MRI. Women without an antenatal suspicion of accreta (despite being histopathologically diagnosed), women with an antenatal suspicion of accreta but it being histopathologically confirmed as nonaccreta, women who declined to participate in this study, women who could not be reached for follow-up, and women who received conservative treatment (did not undergo hysterectomy or placenta left in situ) were excluded from this study. The flowchart of the inclusion process is shown in Figure 1.

### 2.2. Data Collection

Archived patient files and the hospital information system were reviewed retrospectively and patients with suspected PAS in the antenatal period were found. Data including maternal age, body mass index (BMI), gravida and parity, number of previous cesarean deliveries and previous uterine surgeries (myomectomy/curettage), gestational age at birth, anesthesia type, skin and uterine incision types, hysterectomy type, maternal tissue injury, blood product transfusions, surgical duration (defined as minutes from skin incision to skin closure), intraoperative complications, duration of intensive care unit (ICU) stay (days), hospital stay (days), opioid use (morphine equivalents), wound complications, maternal death, fetal/neonatal complications, and APGAR scores were recorded. Cosmetic results of the incision types (satisfactory or dissatisfactory) and postoperative complications were obtained by a telephone interview with the patient. Women with complaints were examined and data were recorded.

### 2.3. Preoperative Management

Unless there was an emergency medical indication, the time of delivery was planned between 34 + 0–35 + 6 weeks. One course of betamethasone therapy was administered for fetal lung maturation in deliveries before 34 weeks. Anti-D immunoglobulin was given to RhD– patients with antenatal hemorrhage. In the preoperative period, patients were informed about skin incisions, hysterectomy, tubal ligation, intestinal injuries, urinary and other organ injuries, blood transfusion, risk of vesicovaginal fistula, anesthesia techniques, risks, and maternal or neonatal ICU requirement, and written consent was obtained. A total of 5 ES and 5 FFP preparations were made for each patient. Except for emergencies, all patients fasted for 8 h preoperatively, no bowel preparation was made, maternal comorbidities were optimized with appropriate drugs (DM, HT, anemia), and prophylactic antibiotics were administered 1 h before the surgery.

### 2.4. Intraoperative and Postoperative Management

All patients underwent surgery in the low lithotomy position. A Foley catheter was placed in the bladder. The skin was disinfected with a povidone-iodine solution 3 times, the vagina was wiped with povidone-iodine, and the patient was covered with a sterile drape. General anesthesia was administered to all patients. The surgical duration was measured from the first skin incision to the last skin suture placement for both techniques.

Surgical Procedures: All surgeons were experienced in retroperitoneal dissection, ureterolysis, internal iliac artery ligation, and all types of abdominal and uterine incisions. Conditions such as placenta localization, PAS grade, previous C/S number, or necessity of emergency delivery did not affect the choice of skin incisions. The selection of the skin incision type (Pfannenstiel or midline) and uterus incision type was based on the surgeon’s preference.

### 2.5. Types of Incisions

#### 2.5.1. Pfannenstiel Group

A Pfannenstiel incision was used to enter the abdomen. A hysterotomy was performed with a transverse incision at least 3 cm above the Kerr incision, targeting the cranial edge of the placenta in the uterus. Although the upper edge of the placenta was targeted, all fetuses were removed by passing through the placenta. After the cord was clamped, the first assistant compressed the lower uterine segment to reduce hemorrhage and the surgeon performed a hysterectomy. The rectus fascia was closed with a size 0 or 1 braided coated polyglactin 910 violet suture, continuously, at 1 cm intervals without locking, without applying excessive tension, and the skin was closed in a subcuticular manner with a size 3-0 polyglactin 910 suture. 

#### 2.5.2. Midline Group

A midline incision (lower midline or lower and upper midline) was used to enter the abdomen. A hysterotomy was performed with a fundal vertical incision. After the fetus was removed, the umbilical cord was clamped and placed in the uterine cavity, the myometrium was sutured, and a hysterectomy was performed. The rectus fascia was closed with a size 1 monofilament polydioxanone violet suture, continuously, without locking, and the skin was closed with a size 2-0 polypropylene suture with the mattress technique.

In both groups, ureteral integrity and peristalsis and bladder injury were evaluated; if the subcutaneous tissue thickness was ≥2 cm, it was approximated with absorbable sutures. A scalpel was used for skin and subcutaneous tissue. No patient underwent endovascular intervention for hemorrhage control.

Postoperative Management: Fluid replacement, antiemetics, and analgesics were effectively utilized. Unless there was a bowel injury, oral intake was initiated at the sixth hour postoperatively, and LMWH was initiated as soon as hemostasis was achieved. The patients were mobilized at the sixth hour postoperatively and if there was no urinary injury, the Foley catheter was removed after mobilization.

### 2.6. Ethical Considerations

This study was approved by Akdeniz University, Faculty of Medicine, Ethics Committee, with Approval No. 147 and the Date of 16 March 2022.

### 2.7. Statistical Analysis

The statistical analysis was performed using IBM SPSS Statistics version 25.0 (IBM Corp., Armonk, NY, USA). The independent two-sample *t*-test was used to analyze the difference in continuous variables between the two groups when the data conformed to normal distribution. The Pearson Chi-Square test and Fisher’s exact test were used to evaluate relationships between categorical variables and the Mann–Whitney *U* test was used when the data were not normally distributed. Descriptive statistics are presented as a number (percentage), mean (±standard deviation), and median (minimum–maximum); *p*-value < 0.05 was accepted as statistically significant.

## 3. Results

A total of 67 consecutively pregnant women with suspected PAS antenatally were enrolled in this study. Figure 1 shows the inclusion and exclusion criteria.

A Pfannenstiel skin incision was used in 49 (73.1%) women, while a midline skin incision was used in 18 (26.9%) women. Conditions such as antepartum hemorrhage, preoperative hemorrhage, and emergency surgery were more common in the Pfannenstiel group (*p* < 0.05 for all). Except for pre-existing conditions such as hypothyroidism, hyperthyroidism, GDM, fetal macrosomia, polyhydramnios, and multiple pregnancies, the other antenatal characteristics were similar in both groups. Demographic data are presented in Table 1.

The total duration of surgery was similar in both groups, 119 min in the Pfannenstiel group and 168 min in the midline incision group (*p* = 0.551). Hypogastric artery ligation was not performed in any of the women in the midline group (0%). In the Pfannenstiel group, five (10.2%) women required relaparotomy within 24 h, whereas in the midline group, none (0%) required relaparotomy. The length of the hospital stay was comparable in both groups (*p* = 0.678). In the histopathological evaluation, 10 (55.6% of cases) in the midline group and only 3 (6.1%) in the Pfannenstiel group were diagnosed with percreta, whereas bladder injury was more common in the Pfannenstiel group (36.7%). A total of two (11.1%) wound site infections occurred in the midline group, while none were in the Pfannenstiel group. Dissatisfaction with the incision site was observed in six (33.3%) cases in the midline group and in one (2%) case in the Pfannenstiel group. Sexual problems due to vaginal dryness or the cosmetic appearance of the incision were also more common in the midline group than in the Pfannenstiel group (27.8 and 6.1%, respectively). Comparisons of intraoperative and postoperative findings and complications by skin incision type are presented in Table 2. 

The median values of the RBC units used in the intraoperative and postoperative periods were 4 (0–22) and 2.5 (0–7) in the Pfannenstiel and the midline groups, respectively. Similarly, in the Pfannenstiel group, 26 (53.1%) cases received more than 4 units of packed RBCs [in the midline group, 6 (33.3%) cases]. Crystalloid and colloid replacements were comparable in both groups (*p* > 0.05 for all). Table 3 gives information about the blood products used intraoperatively and postoperatively. 

Preoperative and postoperative Hb, Htc, and WBC values, and the differences between values, were similar in both groups, as shown in Table 4. 

The mean duration from incision to delivery was 8.6 min in the Pfannenstiel group and 7.7 min in the midline group. The number of neonates requiring NICU admission was 17 (34.7%) in the Pfannenstiel group and 4 (22.2%) in the midline group (*p* = 0.329). Table 5 shows that there were no differences in neonatal outcomes by skin incision types. The outcomes of the current study, as well as the two comparable studies [14,15] available in the literature, are presented in Table 6.

## 4. Discussion

In this study, two different surgical techniques (transverse skin and uterine incision vs. midline skin and classic uterine incision) were compared with respect to surgical outcomes, complications, and cosmetic results in patients with Placenta Accreta Spectrum Disorders. The total duration of surgery was longer in the midline incision group while this difference was not statistically significant. Moreover, a tenth of patients with a transverse skin incision underwent relaparotomy and a third of patients experienced more bladder injury while there was no laparotomy in the midline incision group. Moreover, though not significant, median units of RBCs administered and the percentage of patients receiving > 4 units of packed RBCs were higher in the transverse skin incision group compared to the transverse incision group. Cosmetic dissatisfaction and sexual problems were more prevalent in the midline group.

PAS is a life-threatening situation, which should be evaluated and surgically treated in highly specialized centers with an optimal setting and infrastructure for such a complicated obstetric scenario. Complications such as life-threatening hemorrhage; transfusion-related complications; severe coagulopathy; bladder, ureter, and bowel injuries; relaparotomy; and maternal death are frequently observed with PAS surgery [12,16]. The primary goal of surgery in PAS cases is to avoid massive hemorrhage and tissue injury and ensure the safe delivery of the fetus [8,9,12]. Maternal mortality and morbidity are reduced when delivery is performed in a center of excellence by a multidisciplinary care team [6,8,17]. There is no standardized treatment option for PAS, including an optimal abdominal or uterine incision type, and the consensus opinion concludes that the type of skin incision should be left to the surgical team [6]. There are limited data regarding the effect of abdominal (transverse or midline) incisions on perioperative and postoperative parameters. To our knowledge, this is the first systematic study evaluating the effect of skin incision types (Pfannenstiel and midline) on perioperative and postoperative parameters with a single-center experience.

It is important to keep the surgical duration short to reduce complications related to surgery or anesthesia [18]. In a previous report evaluating the effect of different skin incision types (vertical or transverse) in emergency cesarean sections, surgical outcomes were comparable in two groups but transverse incision slightly increased the surgical duration by 1–2 min [19]. In another study comparing transverse and vertical skin incisions for elective cesarean section hysterectomy, the surgical duration was shorter in the transverse group (180 min versus 238 min), whereas estimated blood loss, intraoperative complications, length of hospital stay, and postoperative opioid use were similar between the groups [13]. In our cohort, although not statistically significant, the mean surgical duration was nearly 50 min shorter in the Pfannenstiel group compared to the midline group. This could be attributed to access to the abdominal cavity and also removal of the fetus by transverse skin and uterine incision. By this way, transplacental incision is performed, which, in contrast, could lead to more blood loss and also loss of tissue dissection planes. In the course of the surgery, loss of clear tissue dissection planes could also lead to a higher incidence of bladder injury in the transverse skin incision group. Conversely, in the midline incision group, more meticulous dissection is carried out, particularly in the formation of a bladder flap, which takes considerable time and effort but with less blood loss and a lower incidence of bladder injury.

The abdominal incisions must allow sufficient access to the uterus. The midline incision is recommended by most authors in PAS surgery because it allows us to perform a hysterotomy above the upper edge of the placenta and to access anatomical structures more easily [8,20,21]. In cases of massive hemorrhage, a midline skin incision provides easier access to hypogastric artery ligation. However, evidence suggests that a Pfannenstiel incision can also be utilized for hypogastric artery ligation [22]. Although midline laparotomy provides a wider area for access to retroperitoneal spaces, in our Pfannenstiel group, 30 (61.2%) women underwent hypogastric artery ligation, whereas none were performed in the midline group. One can assume that hypogastric artery ligation would lead to a reduction in hemorrhage. However, in our Pfannenstiel group, 5 out of 49 women (10.2%) required relaparotomy within 24 h due to ongoing hemorrhage (none in the midline group), which all had hypogastric artery ligation during their initial operation. Midline skin and fundal uterine vertical incision has several advantages over transverse incisions such as wider surgical space, avoidance of transplacental incision, and easier access to vesicouterine space in order to create a bladder flap. In our study, the use of a midline incision in both the skin and uterus could lead to better control of bleeding, finer dissection, and easier access to retroperitoneal structures, which all prevented relaparotomy in this high-risk patient cohort. 

Although a number of patients were diagnosed with placenta accreta in pathologic evaluation, all patients included in this study had a provisional diagnosis of placenta percreta in the antenatal period and during the cesarean section. Placental tissue invading either the uterine serosa, bladder wall, or parametria and abnormal macroscopic findings such as significant hypervascularity and multiple vessels crossing uterine serosa and placental bulge were all observed. In our cohort, 13 women (19.4%) were diagnosed with placenta percreta (confirmed through pathological review). Among them, 10 (76.9%) had a midline skin incision and 3 (23.1%) had a Pfannenstiel incision for surgery.

A Pfannenstiel abdominal incision is expected to be superior to a midline incision with respect to postoperative pain. In a systematic review, higher opioid use was observed in patients who underwent midline skin incisions and it was concluded that a midline abdominal incision is more painful than transverse incisions [23,24]. However, these beneficial effects of Pfannenstiel incisions were not observed in our cohort (*p* = 0.470).

Surgical site infection is a frequently encountered complication in PAS surgery, with a reported prevalence rate ranging from 18% to 32% [12]. In a retrospective observational study, the rate of surgical site infection after transverse skin incision was significantly lower than for midline skin incision (16.8% vs. 27.6%, *p* = 0.04) [25]. On the other hand, the POVATI study reported a higher incidence of surgical site infections after transverse skin incision compared with midline skin incision [26]. In our study, in two groups, the surgical site infection rate was comparable.

Cosmesis is an important factor to consider when choosing an incision. Langer’s lines run horizontally across the skin of the anterior abdominal wall. These lines indicate the direction of minimal tension in the skin, promoting optimal wound healing. Therefore, it is expected that an incision parallel to Langer’s lines will result in minimal cosmetic damage. Transverse incisions are situated along the Langer’s lines of the skin and numerous studies have demonstrated that transverse incisions are cosmetically more satisfactory compared to midline incisions [23,27,28]. Consistent with the previous studies, one (2%) woman in the Pfannenstiel group and six (33.3%) women in the midline group expressed dissatisfaction with the incision site (*p* = 0.001). In previous reports, transverse incision had fewer complications, shorter operation time, less of a blood product requirement, and more cosmetic satisfaction than midline incision [14]. Pfannenstiel incision may be superior to a midline incision in terms of cosmetic concerns. Regarding the additional complaints, three (6.1%) women in the PI group and five (27.8%) in the MI group experienced vaginal dryness. All eight of these women reported that their symptoms began within the first 6 months following the surgery. 

In previous reports, the time from incision to delivery and short-term maternal and infant outcomes were similar in transverse and vertical incisions [29], and in another study, transverse incisions led to delayed delivery of the fetus and prolonged the surgery time [30]. In a prospective cohort study, midline skin incision provided faster access to the abdomen but did not improve neonatal outcomes [19]. Our neonatal outcomes were comparable in two groups. This may be due to the similar mean time from the beginning of the surgery to the delivery of the fetus. 

There are two studies evaluating the effect of skin incisions on perioperative characteristics in patients with PAS [14,15]. Fertility-sparing surgeries were included in the first multicenter study [14], whereas those patients with uterus preservation were excluded in the second study [15]. These two studies [14,15] reported shorter total operative time among participants in the transverse group. The study conducted by Soyer et al. [14] revealed a higher incidence of bladder injuries and relaparotomy in the vertical group. In contrast, though not significant, in the current study, bladder injury rates were higher in the transverse incision group and there was no relaparotomy in the midline incision group. Furthermore, in all three studies, the transverse incision yielded superior cosmetic outcomes compared to the vertical incision (Table 6). 

The strength of our study was that the choice of skin incision type was not dependent on patient characteristics such as BMI, previous C/S, placental localization, PAS grade, or gestational age. This limited the number of significant confounders. Moreover, this was a single-center study with a fairly homogenous patient population (hysterectomy patients only, not fertility-sparing patients). 

Our study’s limitations include being a retrospective study with a small sample size, heterogeneity in the PAS grades, and inter-surgeon variability in surgical techniques. The fact that our hospital is a tertiary center with multidisciplinary expertise with an established blood center, ICU, NICU, and surgeons experienced in PAS surgery may limit the generalizability of our findings to other settings. There may be recall bias due to the inclusion of cases spanning from 2010 to 2022.

## 5. Conclusions

Relaparotomy, bladder injury, blood loss, and need for blood transfusion were more prevalent in the Pfannenstiel group, while greater dissatisfaction with the incision was observed in the midline incision group. Other surgical complications and neonatal outcomes were comparable between the two groups. Midline incision seems to be more favorable in patients with Placenta Accreta Spectrum (PAS). Patients may be informed regarding the worse cosmetic outcomes and possible sexual problems related to vaginal dryness when midline laparotomy is planned. But before opting for a Pfannenstiel incision, patients should receive comprehensive information regarding the potential risks of relaparotomy and bladder injury.

## Figures and Tables

**Table 1:** Participants’ Baseline Characteristics by Type of Skin Incision.

Maternal Characteristics	Pfannenstiel Group (n: 49)	Midline Group (n: 18)	*p*-Value
Number of patients, n (%)	49 (73.1%)	18 (26.9%)	
Maternal age (years), Mean (±SD)	33.4 (4.29)	35.2 (4.38)	0.642
Gravida, Mean (±SD)	3.69 (1.44)	3.77 (1.21)	0.574
Parity, Mean (±SD)	2.18 (1.23)	2.11 (1.07)	0.976
Previous C/S, Mean (±SD)	1.91 (0.67)	2.05 (1.20)	0.877
Notable pre-existing condition ª, n (%)	11 (22.4%)	12 (66.7%)	**0.001**
BMI (kg/m²), Mean (±SD)	31.0 (6.03)	29.9 (4.06)	0.694
BMI ≥ 30 kg/m^2^, n (%)	24 (49.0%)	10 (55.6)	0.633
GA at diagnosis, Mean (±SD)	29.1 (4.54)	25.2 (4.22)	0.890
Placenta localization, n (%) Anterior Posterior	41 (83.7%) 8 (16.3%)	13 (72.2%) 5 (27.8%)	0.312
At the last ultrasound examination Previa, n (%) Not previa, n (%)	46 (93.9%) 3 (6.1%)	17 (94.4%) 1 (5.6%)	1.000
Antepartum hemorrhage	21 (42.9%)	2 (11.1%)	**0.020**
GA of the first episode of antepartum hemorrhage (week), Median (min–max)	30 (20–37)	27 (26–28)	0.225
Hospitalized during pregnancy, n (%)	39 (79.6%)	17 (94.4%)	0.264
Number of days of hospitalization during pregnancy, Mean (±SD)	8.5 (9.75)	10 (7.7)	0.165
Reasons for hospitalization, n (%) Preoperative preparation Antenatal hemorrhage DM Remote residence	38 (77.6%) 5 (10.2%) 0 (0%) 6 (12.2%)	7 (38.9%) 1 (5.6%) 8 (44.4%) 2 (11.2%)	NA
Antenatal PPROM, n (%)	4 (8.2%)	0 (0%)	0.211
Preoperative (emergency) hemorrhage, n (%)	14 (71.4%)	0 (0%)	**0.014**
Emergency surgery, n (%)	23 (46.9%)	1 (5.6%)	**0.002**

SD, standard deviation; min, minimum; max, maximum; GA, gestational age; BMI, body mass index; DM, diabetes mellitus; C/S, cesarean section; n, number of patients; PPROM, preterm premature rupture of membranes; NA, not applicable; ª: Hypothyroidism, hyperthyroidism, DM, fetal macrosomia, polyhydramnios, and multiple pregnancies. Note: A *p*-value < 0.05 indicates statistical significance.

**Table 2:** Comparisons of the intraoperative and postoperative Findings and Complications.

	Pfannenstiel Group (n: 49)	Midline Group (n: 18)	*p*-Value
GA at delivery (week), Median (min–max)	35 (24–40)	34 (29–34)	**0.041**
Total time of surgery (minute), Mean (±SD)	119.51 (42.89)	168.66 (47.15)	0.551
Total hysterectomy, n (%) Subtotal hysterectomy, n (%)	47 (95.9%) 2 (4.1%)	16 (88.9%) 2 (11.1%)	0.282
Hypogastric artery ligation, n (%)	30 (61.2%)	0 (0%)	**0.000**
Salpingectomy, n (%)	23 (46.9%)	15 (83.3%)	**0.008**
Bladder injury, n (%)	18 (36.7%)	3 (16.7%)	0.117
Another tissue injury (ureter, bowel), n (%)	0 (0%)	0 (0%)	NA
Other surgeons present at surgery (urologist), n (%)	5 (10.2%)	3 (16.7%)	0.672
Number of patients with drainage tube, n (%)	44 (89.8%)	17 (94.4%)	0.555
Drainage tube days, Median (min–max)	3 (0–8)	2 (0–9)	0.692
Admission to ICU, n (%)	17 (37.7)	4 (22.2%)	0.329
Days in ICU, Median (min–max)	1 (1–8)	1 (1–1)	0.635
Number of patients using opiate, n (%)	28 (57.1%)	15 (83.3%)	0.470
Opiate dose (mg), Median (min–max)	100 (50–100)	100 (50–200)	0.761
Relap, n (%)	5 (10.2%)	0 (0%)	0.159
Relap time (after first surgery) (hours), Mean (±SD)	10.2 (5.6)	-	NA
Length of hospital stay (days), Median (min–max)	5 (3–18)	5 (2–25)	0.678
Postoperative complications, n (%) None Wound infection Cuff hematoma	48 (98%) 0 (0%) 1 (2%)	16 (88.9%) 2 (11.1%) 0 (0%)	0.052
Pathology report, n (%) Accreta Increta Percreta	43 (87.8%) 3 (6.1%) 3 (6.1%)	8 (44.4%) 0 (0%) 10 (55.6%)	**0.000**
Dissatisfaction with the incision, n (%)	1 (2%)	6 (33.3%)	**0.001**
Vaginal dryness after surgery, n (%)	3 (6.1%)	5 (27.8%)	**0.028**

SD, standard deviation; min, minimum; max, maximum; GA, gestational age; n, number of patients; ICU, intensive care unit; Relap, relaparotomy; NA, not applicable; Note: A *p*-value < 0.05 indicates statistical significance.

**Table 3:** Blood product transfusions intraoperatively and postoperatively.

	Pfannenstiel Group (n: 49)	Midline Group (n: 18)	*p*-Value
Number of patients receiving RBC, n (%)	45 (91.8%)	14 (77.8%)	0.196
Units of RBC, Median (min–max)	4 (0–22)	2.5 (0–7)	0.056
Patients at >4 units of packed RBCs transfused, n (%)	26 (53.1%)	6 (33.3%)	0.152
Number of patients receiving FFP, n (%)	32 (65.3%)	13 (72.2%)	0.593
Units of FFP, Median (min–max)	2 (0–17)	1 (0–7)	0.426
Crystalloid (mL), Median (min–max)	3000 (1000–6000)	3000 (2000–5000)	0.364
Colloid (mL), Median (min–max)	500 (0–1000)	500 (0–500)	0.128

RBC, red blood cell; FFP, fresh frozen plasma; mL, milliliter; n, number of patients; Note: A *p*-value < 0.05 indicates statistical significance.

**Table 4:** Changes in laboratory parameters.

	Pfannenstiel Group (n: 49) Mean ± SD	Midline Group (n: 18) Mean ± SD	*p*-Value
Hb (g/dL) Preoperative Postoperative Difference	11.03 ± 0.98 8.50 ± 1.36 2.53 ± 1.70	10.72 ± 1.25 8.84 ± 1.30 1.87 ± 1.60	0.593 0.483 0.689
Htc (%) Preoperative Postoperative Difference	33.18 ± 3.27 25.26 ± 3.72 7.92 ± 4.78	32.31 ± 3.13 26.30 ± 3.79 6.00 ± 4.76	0.852 0.808 0.732
WBC (10^3^/mL) Preoperative Postoperative Difference	9530 ± 2814 12,940 ± 5828 −3410 ± 5740	9207 ± 2423 12,462 ± 3318 −3255 ± 4294	0.886 0.310 0.457
Platelet (10^3^/mL) Preoperative Postoperative Difference	219.61 ± 73.88 174.59 ± 73.56 45.02 ± 80.28	278.54 ± 59.88 134.54 ± 28.17 144.04 ± 71.33	0.429 0.738 **0.001**

SD, standard deviation; n, number of the patients; Hb, hemoglobin; Htc, hematocrit; WBC, white blood cell. Difference: Preoperative value (−) postoperative value; Note: A *p*-value < 0.05 indicates statistical significance.

**Table 5:** Neonatal outcomes in women with Placenta Accreta Spectrum treated with Pfannenstiel or a midline skin incision.

	Pfannenstiel Group (n: 49)	Midline Group (n: 18)	*p*-Value
Delivery time (minute), Mean (±SD)	8.6 (0.8)	7.7 (1.06)	0.859
Birth weight (grams), Mean (±SD)	2538 (775)	2587 (455)	0.077
APGAR 1 min, Median (min–max) APGAR 5 min, Median (min–max)	8 (0–9)9 (0–10)	7 (1–9)8.5 (6–10)	0.417
Admission to NICU, n (%)	17 (34.7%)	4 (22.2%)	0.329
Days in NICU (days), Median (min–max)	1 (1–8)	1 (1–1)	0.635
Reason for admission to NICU Respiratory distress Infection NEC, IVH, jaundice	11 (22.4%) 4 (8.2%) 4 (8.2%)	9 (50%) 1 (5.6%) 1 (5.6%)	0.065

NICU, neonatal intensive care unit; n, number of patients; NEC, necrotizing enterocolitis; IVH, intraventricular hemorrhage; Note: A *p*-value < 0.05 indicates statistical significance.

**Table 6:** Comparison of the Baseline Characteristics and Intraoperative and Postoperative Findings of the Participants.

Studies		Number of Patients, n (%)	Previous C/S	Antepartum Hemorrhage	Preoperative (Emergency) Hemorrhage, n (%)	Emergency Surgery (%)	GA at Delivery (Week)	Total Time of Surgery (Minute)	Hysterectomy	Bladder Injury, n (%)	Hypogastric Artery Ligation, n (%)	Relaparotomy, n (%)	Length of Hospital Stay (days)	Dissatisfaction with the Incision, n (%)
Soyer-Çalışkan et al. [14]	Transverse Group	81 (69.8%)	2 (1–4) ^b^				35.46 (1.98) ^a^	91.23 (16.53) ^a^	66 (81.5%)	9 (11.1%)		0 (0%)	5 (3–8) ^b^	
	Vertical Group	35 (30.2%)	2 (1–6) ^b^				34.23 (2.82) ^a^	100.44 (18.35) ^a^	29 (82.9%)	13 (37.1%)		2 (5.7%)	4 (3–8) ^b^	
	*p*-Value		**0.014**				**0.024**	**0.010**	**0.860 ^c^**	**0.001**		**0.030**	0.111	
Akbaba [15]	High Transverse Group	24 (54.5%)	2.1 (0.7) ^a^				34.4 (3.5) ^a^	101.5 (8.2) ^a^	24 (100%)				5.5 (1.1) ^a^	3 (12.4%)
	Vertical Group	20 (45.5%)	1.9 (0.7) ^a^				34.3 (3.7) ^a^	129.8 (7.7) ^a^	20 (100%)				8.0 (1.1) ^a^	13 (65%)
	*p*-Value		0.23				0.96	**0.04**					**0.03**	**0.01**
Our Study	Pfannenstiel Group	49 (73.1%)	1.91 (0.67) ^a^	21 (42.9%)	14 (71.4%)	23 (46.9%)	35 (24–40) ^b^	119.51 (42.89) ^a^	49 (100%)	18 (36.7%)	30 (61.2%)	5 (10.2%)	5 (3–18) ^b^	1 (2%)
	Midline Group	18 (26.9%)	2.05 (1.20) ^a^	2 (11.1%)	0 (0%)	1 (5.6%)	34 (29–34) ^b^	168.66 (47.15) ^a^	18 (100%)	3 (16.7%)	0 (0%)	0 (0%)	5 (2–25) ^b^	6 (33.3%)
	*p*-Value		0.877	**0.020**	**0.014**	**0.002**	**0.041**	0.551	1.000	0.117	**0.000**	0.159	0.678	**0.001**

^a^: Mean (±SD); ^b^: Mann–Whitney U test, median (min–max); ^c^: Fisher’s exact test; SD, standard deviation; min, minimum; max, maximum; GA, gestational age; C/S, cesarean section; n, number of patients; Note: A *p*-value < 0.05 indicates statistical significance.

## Data Availability

The data that support the findings of this study are available on request from the corresponding author. The data are not publicly available due to privacy or ethical restrictions.
